# Prevalence and Severity of Gastrointestinal Symptoms in COVID-19 Patients in Casablanca: A Retrospective Cohort Study

**DOI:** 10.7759/cureus.27815

**Published:** 2022-08-09

**Authors:** Fatima Zahra Belabbes, Meryem Maizi, Nouhaila Belghyti, Ihsane Hmamouchi, Mohamed Khalis, Karim El Aidaoui, Aziza Kantri, Chafik El Kettani, Abdelhamid Naitlhou, Fedoua Rouibaa

**Affiliations:** 1 Gastroenterology and Hepatology, Cheikh Khalifa International University Hospital, Faculty of Medicine, Mohammed VI University of Health Sciences, Casablanca, MAR; 2 Pediatrics, Cheikh Khalifa International University Hospital, Faculty of Medicine, Mohammed VI University of Health Sciences, Casablanca, MAR; 3 Laboratory of Biostatistics, Clinical Research and Epidemiology, Mohammed V University in Rabat. Morocco., Casablanca, MAR; 4 Epidemiology and Public Health, Mohammed VI University of Health Sciences, Casablanca, MAR; 5 Anesthesia and Critical Care, Cheikh Khalifa International University Hospital, Faculty of Medicine, Mohammed VI University of Health Sciences, Casablanca, MAR; 6 Anesthesia and Critical Care, Cheikh Khalifa International University Hospital, Casablanca, MAR; 7 Internal Medicine, Cheikh Khalifa International University Hospital, Mohammed VI University of Health Sciences, Casablanca, MAR

**Keywords:** digestive symptoms, severe covid-19, covid-19, gastro-intestinal, sars-cov-2

## Abstract

Background

The novel severe acute respiratory syndrome coronavirus 2 (SARS-CoV-2), responsible for the coronavirus disease 2019 (COVID-19), is behind the current pandemic. At the start of the pandemic, gastrointestinal symptoms initially described as rare were reported, but their spread to other countries increased rapidly. This study aimed to determine the prevalence of digestive symptoms among COVID-19 patients and to assess the correlation between these symptoms and disease severity.

Methods

This retrospective observational study was conducted in the Cheikh Khalifa University Hospital of Casablanca, Morocco. Patients were divided into two groups based on the presence or absence of gastrointestinal symptoms upon initial assessment and hospital admission.

Results

A total of 154 patients were included in this study from March 21 to April 26, 2020. The mean age of patients was about 48.5 (± 20.0) years, and 85 (55.2%) of them were men. In our population, 8.17% of patients had toxic habits. Digestive symptoms were present at admission in 30% of our patients. The most frequent digestive symptoms were diarrhea (15%), abdominal pain (5.6%), vomiting (5%), and anorexia (3.1%). We found a significant difference in COVID-19 patients with digestive symptoms and toxic habits contrary to all other comorbidities. Neurologic symptoms were significantly associated (p=0,004) with digestive symptoms in 50%.

Conclusion

In this study, we found that digestive symptoms were present in 22.64% of patients diagnosed with COVID-9. The clinician must know the different digestive symptoms to evoke the diagnosis and take charge of the patient early.

## Introduction

In December 2019, a new virus emerged In China and created a pandemic alert worldwide: COVID-19. In Morocco, the first case of this disease was declared on March 2, 2020, and the first death on March 11, 2020. This virus is transmitted through respiratory droplets or close contact with an infected individual. Several studies have been conducted to study the severity factors of COVID-19. Still, no study in Morocco has been carried out about digestive symptoms and COVID in our context according to our knowledge. The symptoms induced by this virus are mainly characterized by respiratory symptoms [1]. However, gastrointestinal symptoms have also been reported and seem to have an important part in the process. This can be explained by the fact that the angiotensin-converting enzyme 2 (ACE-2) receptor is essential for cells infected by COVID-19 [2]. It has been shown to be highly expressed in the digestive system, particularly in the upper esophagus and epithelial cells and in the absorptive enterocytes of the ileum and colon [3]. The main aim of this study was to determine the prevalence of digestive symptoms among COVID-9 patients and to assess the correlation between these symptoms and disease severity.

## Materials and methods

Study design

This study is a single-center retrospective observational study conducted in the Cheikh Khalifa University Hospital, Casablanca, Morocco, from March 21 to April 26, 2020.

Participants and eligibility criteria

The participants were COVID-19 patients who met the inclusion criteria of this study. The inclusion criteria consisted of adult patients (≥18 years old) diagnosed with confirmed COVID-19 infection. Patients were divided into two groups based on the presence or absence of gastrointestinal (GI) symptoms upon initial assessment and hospital admission.

A laboratory-confirmed case of COVID-19 was defined as a positive pharyngeal swab test using real-time reverse transcription PCR (RT-PCR) for SARS-CoV-2 [1], performed at the National Laboratory Research (LNR) affiliated to the University Mohammed VI of Health Sciences. Negative RT-PCR and/or normal biologic and radiologic exams were excluded.

Suspected cases of COVID

The suspicious case presents at the time of admission with fever, dyspnea, cough [3,4], a crazy-paving pattern, and/or ground glass opacities on chest computed tomography (CT) scan [5], and biological stigma of inflammation including leukopenia, lymphocytopenia, thrombocytopenia, elevated C-reactive protein (CRP), serum ferritin, and lactate dehydrogenase [6,7].

CT scan

Chest CT scans were performed in our radiology unit and interpreted following the COVID-19 Reporting and Data System (CARDS), assessment established by the Dutch Radiological Society (NVvR) [8].

Severe form

Severely ill patients were defined as those admitted to the ICU who had one of the following signs: respiratory rate > 30 breaths/min, oxygen saturation < 93% on room air, acute respiratory distress syndrome (ARDS), or requiring mechanical ventilation. Non-severe patients were those with mild or moderate forms of COVID-19, according to the World Health Organization interim guidance now data [9].

Toxic habit was defined by alcohol consumption and cigarette smoking. Hepatic injury was defined by elevated liver enzymes: aspartate transaminase ≥ 34 UI/L and alanine transaminase ≥ 55 UI/L.

Data collection

Our data were collected using an electronic data system DxCare and Laboratory information management system (DxCare SIH-HCK version 7.7-7p059 (Dedalus, France) and LIMS SGL-HCK version 11.4.3-26122018 (eNOVA). For included patients who had already left the hospital with missing data, direct communication by phone call was retrospectively made in order to gain in-depth medical history. For each patient, we collected data including sociodemographic characteristics (age, sex, etc.), coexisting conditions, clinical data including digestive symptoms (nausea, vomiting, diarrhea, abdominal pain, anorexia, and hepatic injury), laboratory data, and radiologic data.

Statistical analysis

Data analysis was performed using SPSS Version 20 software (SPSS Inc., Armonk, NY, USA). The patients were divided into two groups according to the presence or absence of GI symptoms, and a comparison was made according to the severity of the disease. Continuous variables were presented as mean and standard deviation and categorical variables as number and percentage. To compare the groups, we used Student’s t-test for the variables with a normal distribution and the Mann-Whitney test for non-parametric variables. Proportions were compared between groups using the chi-square test or Fisher’s exact test. In all statistical analyses, p-values were two-sided and considered statistically significant if lower than 0.05.

## Results

We registered 154 patients in our study from March 21 to April 26, 2020. Among them, 36 had digestive symptoms on admission and 118 did not. Out of the 36 cases with digestive symptoms, diarrhea was the most frequent symptom, followed by abdominal pain, vomiting, anorexia, constipation, and nausea. Almost a quarter of patients had liver enzymatic elevation on admission (Table 1).

**Table 1:** Digestive symptoms ^1^Expressed in frequency (%). ^2^Expressed in median [interquartile range] AST, aspartate transaminase; ALT, alanine transaminase

Digestive manifestations	N=154
Abdominal pain	9 (5.8%)
Diarrhea^1^	24 (15.6%)
Vomiting^1^	8 (5.2%)
Nausea^1^	2 (1.3%)
Anorexia^1^	5 (3.2%)
Constipation^1^	3 (1.9%)
Total^1^	36 (23.4%)
AST^2^	23 [18.75–32.250]
ALT^2^	22 [15–36]

Demographic and clinical characteristics

Epidemiological Characteristics

The mean age of our population was 48.5 (±20) years, with a sex ratio of 1.23. In our population, 28.6% have blood hypertension, 14.9% have diabetes mellitus, 9.7% have cardiovascular disease, 9.1% have lung disease, 8.4% have toxic habits, 4.5% have autoimmune disease, 3.2% have chronic liver disease, and 2.6% have kidney failure. Moreover, a significant difference has been found in COVID-19 patients with digestive symptoms and toxic habits contrary to all other included comorbidities (Table 2).

**Table 2:** Epidemiological characteristics

Characteristics	All cases (n=154)	With digestive symptoms (n=36)	Without digestive symptoms (n=118)	p-Value
Age (years)				
>60	52 (33%)	11 (21.2%)	41 (78.8%)	0.89
<60	102 (66.2%)	25 (24.5%)	77 (75.5%)	0.642
Sex				
Male	85 (55.2%)	19 (52.8%)	66 (55.9%)	0.739
Comorbidities				
Any		17 (47.2%)	56 (47.5%)	0.980
Hypertension	44 (28.6%)	11 (30.6%)	33 (28.0%)	0.826
Diabetes	23 (14.9%)	6 (16.7%)	17 (14.4%)	0.816
Cardiovascular disease	15 (9.7%)	4 (11.1%)	11 (9.3%)	0.819
Lung disease	14 (9.1%)	4 (11.1%)	10 (8.5%)	0.769
Autoimmune disease	7 (4.5%)	3 (8.3%)	4 (3.4%)	0.399
Chronic liver disease	5 (3.2%)	3 (8. 3%)	2 (1.7%)	0.126
Kidney failure	4 (2.6%)	2 (5.6%)	2 (1.7%)	0.384
Toxic habits	13 (8.4%)	7 (19.4%)	6 (5.1%)	0.02

Clinical and Laboratory Characteristics

As shown in Table 3, 36 patients expressed digestive symptoms on admission and 118 did not. Respiratory symptoms and fever were the most common clinical features on admission in patients with GI symptoms, with a proportion of 66, 7%, and 50%, respectively. Additionally, neurologic symptoms were significantly associated (p=0.004) with digestive symptoms in 50%. There was no significant association between prognostic markers of COVID-19 severity and GI symptoms.

**Table 3:** Clinical and laboratory characteristics ^1^Expressed in frequency (%). ^2^Expressed in median [interquartile range]. AST, aspartate transaminase; ALT, alanine transaminase

Characteristics	All cases (n=154)	Cases with digestive symptoms (n=36)	Cases without digestive symptoms (n=118)	p-Value
Clinical features				
Non-digestive symptoms^1^	118	36 (94.4%)	81 (68.6%)	0.002
Fever^1^	66 (42.9%)	18 (50%)	48 (40.7%)	0.322
Neurological symptoms^1^	47 (30.5%)	18 (50%)	29 (24.6%)	0.004
Respiratory symptoms^1^	87 (56.5%)	24 (66.7%)	63 (53.4%)	0.16
Laboratory findings				
AST^2^ (U/L)	23 [18.75–32.250]	33.97	33.13	0.909
ALT^2 ^(U/L)	22 [15–36]	27.02	30.33	0.464
Hemoglobin ^2^ (g/dL)	13.9 [12.9–15.1]	14.12	13.72	0.194
White blood cell^2 ^(x10/L)	6,365 [4,882.5–7,537.5]	6656.76	6,617.72	0.943
Lymphocytes	1,500 [1,020–2,110]	1874.55	1,523.26	0.035
Platelets^2 ^(x10/L)	231,000 [180,000–280,000]	234,882.35	247,914.98	0.483
C-reactive protein^2 ^(mg/L)	9 [1.9–71 ]	45.33	46.76	0.512
Serum ferritin^2 ^(ug/L)	132 [49.5–398]	375.80	316.88	0.607
Lactate dehydrogenase^2 ^(U/L)	227.5 [177.5–278.5]	246	249.04	0.883
D-dimer^2 ^(µg/L)	517 [302.62–887.075]	1075.64	3119.47	0.512

No significant difference was found concerning the duration of hospitalization, clinical-biological evolution, and mortality between the two groups (Table 4).

**Table 4:** Outcomes of patients with and without digestives symptoms ^1^Expressed in mean ± standard deviation

Characteristics	All cases (n=154)	Cases with digestive symptoms (n=36)
Hospitalization period^1^	12.016±6	10.107±6.5
Hospital unit		
Intensive care unit	36 (23.3%)	7 (19.4%)
Resuscitation rooms	118 (76.6%)	29 (80.6%)
Death	15 (9.7%)	2 (5.6%)
Biological recovery	35 (22.7%)	9 (25.7%)
Clinical recovery	71 (46.1%)	16 (22.5%)
Non-recovery	5 (3.2%)	3 (60%)

## Discussion

This study was conducted in Casablanca to deal with COVID-19. The patients were tested, and if proved to be COVID-19 positive by PCR, then they were admitted to a hospital designed and equipped for isolation of PCR-positive patients and treatment of symptomatic patients. In this single-center retrospective study, we have studied the prevalence of GI manifestation and the correlation between these symptoms and disease severity.

The end of 2019 was marked by the emergence of a new coronavirus, SARS-Cov-2 [10,11,12,13], initially found in the city of Wuhan in China. SARS-CoV2 -2 is responsible for polymorphous digestive symptomatology that may precede the onset of respiratory symptoms [11,12]. Subsequent studies reported digestive symptoms in COVID-19 patients with varying frequencies ranging from 4.7% to 62% [14,15].

In our study, 36 had digestive symptoms on admission and 118 did not. Moreover, a significant difference has been found in COVID-19 patients with digestive symptoms and toxic habits contrary to all other included comorbidities. Diarrhea was the most frequent symptom, followed by abdominal pain, vomiting, anorexia, constipation, and nausea. In our series, almost a quarter of our patients had a hepatic enzymatic abnormality. Neurologic symptoms were significantly associated (p=0.004) with digestive symptoms in 50%.

SARS-Cov-2 is a respiratory virus with a tropism, particularly for the type 2 alveolar cell. The enterocyte has been identified as the target cell of SARS-CoV-2 in the digestive tract. Small intestine enterocytes and colonocytes showed the highest proportions of cells co-expressing ACE-2 receptor and the transmembrane serine protease 2 (TMPRSS2) [16]. Detection of viral ribonucleic acid (RNA) and viral particles in the stool raised the hypothesis of possible fecal-oral transmission of SARS-Cov-2. SARS-CoV-2 infection of enterocytes induces the cytokine response responsible for the inflammatory reaction with structural and functional alterations of enterocytes [17].

GI manifestations have been reported from the first cases of COVID-19. Within our cohort, 36 (23.5%) patients presented with GI symptoms that are lower than the results reported [14,15]. From the start of the epidemic in China, diarrhea was reported in two of six patients in the COVID-19 family cluster in the city [12]. In our study, diarrhea was the most frequent symptom up to 15.6%. In a COVID-19 epidemic context, an infection linked to SARS-CoV-2 should be suspected in case of any febrile diarrhea. Wang et al. found that abdominal pain and anorexia were significantly more common in intensive care unit (ICU) hospital patients than in patients receiving routine care (8.3% vs 0%, p = 0.02 and 66.7% vs 30.4%, p < 0.001, respectively) [18]. Liver damage has been described in 16% to 72.4% of COVID-19 patients, and impaired liver function as assessed by elevated transaminases (32.6%) is the most relevant feature [8]. In our series, almost a quarter of our patients had a hepatic enzymatic abnormality. Digestive hemorrhages remain rare and are reported in some series to be between 3% and 10%. On the limited data available in endoscopy, it would be peptic ulcer disease, erosive gastritis, and sometimes acute colitis [10].

In our study, no significant difference was found concerning the duration of hospitalization, clinical-biological evolution, and mortality between the two groups. The mortality was high in patients without digestive signs. There was no significant association between prognostic markers of COVID-19 severity and GI symptoms. Our results disagree with previous studies that showed that in the presence of GI symptoms, the disease is more severe of disease with more severe pneumonia and hospitalization in intensive care [19]. Considering analyses of studies reporting specific groups or outcomes, patients with severe diseases were more likely to have GI symptoms: 17.1% of severe patients versus 11.8% of non-severe patients [2]. They explained this hypothesis by the increase in viral load in the presence of GI symptoms. Xiao et al. investigated 73 SARS-CoV-2 infected hospitalized patients in China and found that 53.4% of patients tested positive for the virus in the stool ranging from the day [20]. The fecal RNA positivity rate in stool was 48%. This detection rate was more elevated in diarrheal patients. Various studies have reported a raised CRP and lymphopenia in patients with COVID-19, and this is associated with more severe illness and patients requiring longer hospital admissions [5]. GI symptoms are non-specific to COVID-19, but it may slow the recovery of those patients, and digestive symptoms may worsen as they get sicker. Our study has limitations. First, the sample size remains limited, as it is a retrospective design, single-center hospital-based study. We do not have detailed and representative data on the population on the amount of smoking and alcohol consumption during and/or before the period linked to COVID‐19. A bigger study with a large sample would be required to validate the results of this study.

## Conclusions

GI symptoms are of particular significance in patients with COVID-19 because, in contrast to other coronaviruses, they appear early and may worsen during the course of the disease. These patients may be associated with a poor prognosis. The underlying mechanisms of SARS-CoV-2 related GI symptoms need to be clarified in future studies. GI symptoms are common and may be associated with respiratory signs in few cases. They have been reported to be tougher in severe illness. Stool PCR for COVID 19 can be positive, as the SARS-COV-2 remains in loose stools for a long time. Therefore, faecal-oral transmission of COVID is possible.

In this single-center retrospective observational study of 154 patients with COVID-19, neurologic symptoms were significantly associated with digestive symptoms. We could not find a statistically significant association between ICU admission in patients with GI symptoms compared with those without GI symptoms. However, further investigation is warranted to better assess this possible association.
